# Vasopressors for the management of maternal hypotension during cesarean section under spinal anesthesia: A Systematic review and network meta-analysis protocol

**DOI:** 10.1097/MD.0000000000013947

**Published:** 2019-01-04

**Authors:** Choongun Ryu, Geun Joo Choi, Yong Hee Park, Hyun Kang

**Affiliations:** Department of Anesthesiology and Pain Medicine, Chung-Ang University Hospital, Seoul, Republic of Korea.

**Keywords:** anesthesia, cesarean section, hypotension, meta-analysis, spinal, systematic review, vasoconstrictor agents

## Abstract

**Background:**

Spinal anesthesia is the standard technique for elective cesarean section, but the incidence of maternal hypotension in this setting is reportedly about 80%, without any prophylactic management. Vasopressors are the most reliable method for counteracting the hypotension induced by spinal anesthesia. However, studies investigating the effects of vasopressors have yielded inconsistent and debatable results. Therefore, we plan to conduct a systematic review and network meta-analysis to identify the most effective vasopressor to prevent maternal hypotension, and to decrease fetal acidosis in women undergoing spinal anesthesia for elective cesarean section.

**Methods:**

A systematic and comprehensive search to detect all the randomized controlled studies on vasopressors for the management of maternal hypotension during cesarean section under spinal anesthesia will be performed using information in the databases, MEDLINE, EMBASE, the Cochrane Central Register of Controlled Trials (CENTRAL), and Google Scholar, beginning from their inception to October 2018. We will conduct a network meta-analysis to combine the direct and indirect comparisons of the vasopressors. We will use the surface under the cumulative ranking curve (SUCRA) values and rankograms to present the hierarchy of vasopressors. A comparison-adjusted funnel plot will be used to assess the presence of small-study effects. The quality of the studies included will be assessed using the risk of bias tool. All statistical analyses will be performed using Stata SE version 15.0.

**Ethics and dissemination:**

This systematic review and meta-analysis will be published in a peer-reviewed journal. Ethical approval and informed consent are not required, as the study will be a literature review and will not involve direct contact with patients or alterations to patient care.

**Trial registration number:**

The protocol for this review has been registered in the PROSPERO network (registration number: CRD42018111852).

## Abstract 2

Supplemental Digital Content is available in the text

## 1 Introduction

Spinal anesthesia is the recommended standard practice for elective cesarean section. It offers a rapid onset and a reliable surgical condition with a failure rate below 1%.^[1,2]^ However, the risk of maternal hypotension is higher with spinal anesthesia than with epidural anesthesia. This is, because spinal anesthesia results in a rapid sympathetic vasomotor blockade: arteriolar vasodilation and decreased systemic vascular resistance (SVR), that is difficult to titrate.^[3]^ Previous studies have reported incidences of maternal hypotension in this setting by up to 80% in the absence of prophylaxis.^[4–6]^ Maternal hypotension does not only result in unpleasant complications such as nausea and vomiting in the parturient, but also decreases the uteroplacental blood flow, consequently, lead to a low Apgar score and fetal acidosis. These conditions have been correlated to the severity and the duration of hypotension.

Previously, maternal hypotension and fetal outcome were thought to be improved by avoiding aortocaval compression (left uterine displacement) and increasing the blood volume, such as by intravenous fluid loading to increase the venous return, cardiac filling pressure, and cardiac output (CO). These techniques, however, have proven ineffective, and use of vasopressors is the most reliable method for countering the hypotension induced by spinal anesthesia.^[7]^

Vasopressor drugs act on α1-, β1- and β2-adrenoreceptors in the heart and vascular system. The physiological response of these adrenoreceptor agonists depends on the type and location of the receptors. Vasoconstriction is mainly mediated by α1-receptors. However, some vasopressors can also stimulate β1- and/or β2-receptors directly or indirectly, leading to positive inotropic (increasing cardiac contractility) and/or positive chronotropic (increasing heart rate, HR) effects. The complex hemodynamic effects of the various vasoconstrictors depend on the relative stimulation of these adrenoreceptors. Reflex cardiovascular responses to vasopressors, on the other hand, may result in other changes, including the unwanted reflex bradycardia.

As for the fetal acid-base changes, pH and base excess (BE) are considered as important outcome parameters in neonates, but these can also be affected by the choice of vasopressor used to manage the maternal hypotension. Owing to low resistance, the uteroplacental blood flow is influenced by the maternal blood pressure (BP). However, several studies on prophylactic administration of vasoconstrictors have reported conflicting results, particularly with regards to fetal acidosis.

In an early study conducted on pregnant ewes, it was found that metaraminol and methoxamine consistently decreased the uterine blood flow compared to ephedrine. Consequently, ephedrine became the first-line vasopressor used in obstetric anesthesia for decades.^[8]^ However, recent clinical trials have demonstrated that phenylephrine, which has a potent direct α1-effect, decreased the risk of fetal acidosis compared to ephedrine.^[9,10]^ These findings have led to evidence-based changes in present-day obstetric anesthesia practice. The pH and BE values are still within the normal range in many studies, and no difference in the incidence of true fetal acidosis and neonatal morbidities have been reported in systematic reviews of randomized controlled trials (RCTs) of ephedrine versus phenylephrine.^[11,12]^

Phenylephrine is a pure vasoconstrictor, so its use is often associated with reflex bradycardia as described above, and a consequent decrease in CO. Cardiac output is an important component for oxygen delivery to the peripheral tissues, and so, would be more important especially under conditions of fetal hypoxemia during delivery. Although a healthy parturient and the healthy fetus may tolerate the decreased HR and CO, the effects on the fetus whose condition is already compromised has not been fully evaluated.^[13]^ This may heighten the attention to parturients in whom reactive hypertension may be harmful, for instance, in parturients with chronic hypertension or preeclampsia, and those with compromised uteroplacental perfusion. Responding to this emerging information, some investigators have suggested the use of norepinephrine as a potential alternative to phenylephrine. Norepinephrine is not only a potent α1-adrenergic agonist, but also is also a relatively mild β1-agonist, and increases both, the HR and cardiac contractility. So, it might be an effective vasopressor for maintaining the maternal BP, and even the CO, during spinal anesthesia.^[14]^

The ideal vasopressor in the field of obstetric anesthesia would not only maintain maternal hemodynamic stability and prevent maternal complications such as nausea and vomiting, but also have minimal detrimental effects on the uteroplacental blood flow, and the neonatal clinical outcomes. However, an ideal vasopressor has been the topic of controversy and is much debated, as described above briefly, and it has not yet been clearly demonstrated whether the clinical benefits may be outweighed by possible disadvantages.

### 1.1 Review objective

This review will compare and specifically evaluate the most effective vasopressor for simultaneously preventing maternal hypotension (effectiveness), and decreasing fetal acidosis (safety) in women undergoing spinal anesthesia for elective cesarean section. All the RCTs that have compared the benefits and disadvantages of commonly used vasopressors in the field of obstetric anesthesia will be reviewed, and statistical analysis will be performed via network meta-analysis.

## 2 Methods and analysis

Our protocol for systematic review and network meta-analysis (NMA) was developed according to the preferred reporting requirements for systematic review and meta-analysis protocol (PRISMA-P) statement.^[15]^ The protocol for this review was registered with the PROSPERO network (registration number: CRD42018111852). This systematic review and the NMA of vasopressors for the management of maternal hypotension during cesarean section under spinal anesthesia will be performed according to the protocol recommended by the Cochrane Collaboration,^[16]^ and will be reported according to the PRISMA extension for network meta-analysis guidelines.^[17]^

### 2.1 Inclusion and exclusion criteria

We propose inclusion of only RCTs that compare 2 or more vasopressors for the management of maternal hypotension during cesarean section under spinal anesthesia.

The PICO-SD information comprises: patients (P): all parturient receiving cesarean section under spinal anesthesia, intervention (I): use of vasopressors to treat or prevent hypotension in parturients receiving cesarean section, (C): other vasopressors or placebo or no treatment, outcome measures, (O): the primary outcomes are systolic blood pressure measured during the early (0–20 min) and middle (20 min - 1 h) phases of cesarean section, maximum and minimum systolic blood pressure during the caesarean section, incidences of hypertension and hypotension during the caesarean section, incidences of fetal acidosis in the umbilical artery or vein and pH of the umbilical artery or vein. The secondary outcomes are incidences of bradycardia and tachycardia during the cesarean section, incidences of nausea, vomiting, nausea and vomiting after cesarean section, mean arterial blood pressure and HR during the cesarean section, fetal APGAR score, and the study design (SD): RCT.

The exclusion criteria are:

1. review articles, case reports, case-series, letters to the editor, commentaries, proceedings, laboratory science studies, and all other non-relevant studies, and
2. studies that failed to report the outcomes of interest. There will neither be language limitations nor date restrictions in our study.

### 2.2 Information sources

#### 2.2.1 Electronic search

We propose searching MEDLINE, EMBASE, the Cochrane Central Register of Controlled Trials (CENTRAL), and Google Scholar using the search terms related to vasopressors for the management of maternal hypotension during cesarean section under spinal anesthesia. Search terms to be used for MEDLINE and EMBASE are presented in the Appendix (Supplementary File). Two authors will screen the titles and abstracts of the retrieved articles. Reference lists will be imported to Endnote software 8.1 (Thompson Reuters, CA, USA) and duplicate articles removed. Additional but relevant articles will be identified by scanning the reference lists of articles obtained from the original search.

### 2.3 Study selection

The titles and abstracts identified through the search strategy described above will be reviewed independently by 2 investigators. To minimize data duplication due to multiple reporting, papers from the same author, organization or country will be compared. For articles determined to be eligible based on the title or abstract, the full paper will be retrieved. Potentially relevant studies chosen by at least 1 author will be retrieved and the full text evaluated. Articles meeting the inclusion criteria will be assessed separately by 2 authors, and any disagreement will be resolved through discussion. In cases where agreement cannot be reached, the dispute will be resolved with the help of a third investigator. If authors are similar or incidence data are extracted from the same database, the study period will be assessed. If the study period overlaps, only the latest study will be included. A flow diagram for the search and selection process will be developed using the PRISMA guidelines.

### 2.4 Data extraction

Using a standardized extraction form, the following data will be extracted independently by 2 investigators:

1. title,
2. name of first author,
3. name of journal,
4. year of publication,
5. study design,
6. kinds of vasopressors,
7. dose of vasopressors,
8. country,
9. risk of bias,
10. inclusion criteria,
11. exclusion criteria,
12. age,
13. number of subjects,
14. systolic blood pressure measured during the early (0–20 min) and middle (20 min - 1 h) phases of cesarean section.,
15. maximum systolic blood pressure during caesarean section,
16. minimum systolic blood pressure during caesarean section,
17. incidence of hypertension during caesarean section,
18. incidence of hypotension during caesarean section,
19. incidence of fetal acidosis in the umbilical artery or vein,
20. pH of the umbilical artery or vein,
21. incidence of bradycardia during caesarean section,
22. incidence of tachycardia during caesarean section,
23. incidence of nausea after caesarean section,
24. incidence of vomiting after caesarean section,
25. incidence of nausea and vomiting after caesarean section,
26. mean arterial pressure during caesarean section,
27. heart rate during caesarean section, and
28. fetal APGAR score.

If the information is inadequate, attempts will be made to contact the study authors, and additional information requested. If unsuccessful, missing information will be calculated from the available data if possible, or will be extracted from the figure using the open source software Plot Digitizer (version 2.6.8; http://plotdigitizer. sourceforge.net).

The reference list will be divided into 2 halves. Two investigators will complete data extraction, 1 for each half of the reference list. Data extraction forms will be cross-checked to verify accuracy and consistency of the extracted data.

### 2.5 Study quality assessment

The quality of the studies will be independently assessed by 2 investigators using the ‘risk of bias’ tool according to the Review Manager (version 5.3, The Cochrane Collaboration, Oxford, UK). The quality will be evaluated using the following potential sources of bias: sequence generation, allocation concealment, blinding of participants or outcome assessor, incomplete data, and selective reporting. The methodology for each study will be graded as “high”, “low”, or “unclear”, to reflect the risk of bias.^[16]^

### 2.6 Statistical analysis

Ad-hoc tables will be designed to summarize data from the included studies and show their key characteristics and an important question related to the review objectives. After extracting the data, reviewers will determine the feasibility of a meta-analysis.

A multiple treatment comparisons NMA is a meta-analysis generalization method that includes both direct and indirect RCT comparison of treatments. A random-effects NMA based on a frequentist framework will be performed using STATA software (version 15; StataCorp LP, College Station, TX) *mvmeta* with NMA graphical tools developed by Chaimani and colleagues.^[18]^

Before conducting the NMA, we plan to evaluate the transitivity assumptions by examining the comparability of the risk of bias (all versus removing high risks of bias for randomization, allocation concealment, and blinding of outcome assessor), demographics and kinds of vasopressor as a potential treatment-effect modifier across comparisons.

A network plot linking all the included vasopressors will be formed to indicate the kinds of vasopressors, number of parturients under different vasopressors, and the level of pair-wise comparisons. The nodes will show vasopressors being compared, and the edges will show the available direct comparisons among the vasopressors. The nodes and edges will be weighed on the basis of the number of parturients and studies.

Contribution plots present the percent contribution of each estimate in the summary estimate and the entire network. We will display the contribution percentage of each comparison by weighted squares in a contribution plot.

We will evaluate the consistency assumption for the entire network using the design-by-treatment interaction model. We will also evaluate each closed loop in the network in order to evaluate local inconsistencies between the direct and indirect effect estimates for the same comparison. For each loop, we will estimate the inconsistency factor (IF) as the absolute difference between the direct and indirect estimates for each paired comparison in the loop.^[19]^

Mean summary effects with confidence interval (CI) will be presented together with their predictive intervals (PrIs) to facilitate interpretation of the results considering the magnitude of heterogeneity. PrIs provide an interval, which is expected to encompass the estimate of a future study.

A rankogram and cumulative ranking curve will be drawn for each vasopressor. A rankogram plots the probabilities for treatments to assume a possible rank. We used the surface under the cumulative ranking curve (SUCRA) values to present the hierarchy of vasopressors for the primary and secondary outcomes. The SUCRA is a relative ranking measure that accounts for the uncertainty in the treatment order, that is, accounts both for the location and the variance of all relative treatment effects. A higher SUCRA value will be regarded as a better result for individual interventions.^[20]^

A comparison-adjusted funnel plot will be used to assess the presence of small-study effects.^[21]^

### 2.7 Sub-group analysis

Sub-group analysis will be carried out based on the quality of study, when possible.

### 2.8 Sensitivity analysis

Sensitivity analyses will be conducted to evaluate the influence of individual studies on the overall effects estimate by excluding 1 study at a time from the analysis.

## 3 Discussion

The objectives of this systematic review and NMA are to investigate and identify the most effective vasopressor to simultaneously prevent maternal hypotension (effectiveness) and to decrease fetal acidosis (safety) in women undergoing spinal anesthesia for elective cesarean section.

To our knowledge, there are very few systematic reviews and meta-analyses published on this topic.^[10–12]^ However, they are focused either on ephedrine and/or phenylephrine only, even though several vasoconstrictors are used in the field of obstetric anesthesia. Therefore, we felt necessitated to compare all kinds of drugs that have been clinically used for preventing maternal hypotension during cesarean section. Hence, we planned this systematic review and the NMA to summarize, and assess the published evidence to-date. Thus, we can identify the most effective and safest vasopressor for the parturient and the fetus. At the same time, this result would shed light on areas for future research.

We anticipate some potential limitations to our analysis. First, the different doses and injection modalities, that is bolus injection or continuous infusion, of vasoconstrictors could be a source of inter-study heterogeneity. Second, there may be some differences in the incidences of hypotension and reactive hypertension as determined by BP measurement, non-invasive intermittent measurement, or invasive continuous measurement. Third, all incidences may depend on the definition of each measurement outcome; maternal hypotension, reactive hypertension, bradycardia, fetal acidosis, etc. These limitations will be described in the discussion section of our review after a comprehensive analysis.

## Author contributions

CR, GJC, YHP, and HK conceived this study. CR, GJC, and YHP developed the study protocol and will implement the systematic review under the supervision of HK. GJC and HK will plan the statistical analyses for the study and will conduct data analysis. CR and YHP will perform the study search, screening, and extraction of data. GJC and HK will review the work. CR will write the initial draft of our review. All authors provided input to finalize the protocols and refine the manuscript.

**Conceptualization:** Choongun Ryu, Geun Joo Choi, Yong Hee Park, Hyun Kang.

**Data curation:** Choongun Ryu, Geun Joo Choi, Yong Hee Park, Hyun Kang.

**Formal analysis:** Geun Joo Choi, Yong Hee Park, Hyun Kang.

**Funding acquisition:** Hyun Kang.

**Investigation:** Choongun Ryu, Geun Joo Choi, Yong Hee Park, Hyun Kang.

**Methodology:** Choongun Ryu, Geun Joo Choi, Yong Hee Park, Hyun Kang.

**Project administration:** Hyun Kang.

**Resources:** Hyun Kang.

**Supervision:** Hyun Kang.

**Writing – original draft:** Choongun Ryu, Geun Joo Choi, Yong Hee Park, Hyun Kang.

**Writing – review & editing:** Hyun Kang.

Hyun Kang orcid: 0000-0003-2844-5880.

## Supplementary Material

Supplemental Digital Content

## References

[1] Riley ET Cohen SE Macario A Spinal versus epidural anesthesia for cesarean section: a comparison of time efficiency, costs, charges, and complications. Anesth Analg 1995;80:709–12. 7893022 10.1097/00000539-199504000-00010

[2] Fettes PD Jansson JR Wildsmith JA Failed spinal anaesthesia: mechanisms, management, and prevention. Br J Anesth 2009;102:739–48. 10.1093/bja/aep096 19420004

[3] Dyer RA Biccard BM Ephedrine for spinal hypotension during elective caesarean section: the final nail in the coffin? Acta Anaesthesiol Scand 2012;56:807–9. 22780437 10.1111/j.1399-6576.2012.02719.x

[4] Clark RB Thompson DS Thompson CH Prevention of spinal hypotension associated with Cesarean section. Anesthesiology 1976;45:670–4. 984486 10.1097/00000542-197612000-00018

[5] Hall PA Bennett A Wilkes MP Spinal anaesthesia for caesarean section: comparison of infusions of phenylephrine and ephedrine. Br J Anaesth 1994;73:471–4. 7999486 10.1093/bja/73.4.471

[6] Kang YG Abouleish E Caritis S Prophylactic intravenous ephedrine infusion during spinal anesthesia for cesarean section. Anesth Analg 1982;61:839–42. 7125249

[7] Cyna AM Andrew M Emmett RS Techniques for preventing hypotension during spinal anaesthesia for caesarean section. Cochrane Database Syst Rev 2006;Cd002251. 10.1002/14651858.CD002251.pub2 17054153

[8] Ralston DH Shnider SM DeLorimier AA Effects of equipotent ephedrine, metaraminol, mephentermine, and methoxamine on uterine blood flow in the pregnant ewe. Anesthesiology 1974;40:354–70. 4819091 10.1097/00000542-197404000-00009

[9] Cooper DW Carpenter M Mowbray P Fetal and maternal effects of phenylephrine and ephedrine during spinal anesthesia for cesarean delivery. Anesthesiology 2002;97:1582–90. 12459688 10.1097/00000542-200212000-00034

[10] Veeser M Hofmann T Roth R Vasopressors for the management of hypotension after spinal anesthesia for elective caesarean section. Systematic review and cumulative meta-analysis. Acta Anaesthesiol Scand 2012;56:810–6. 22313496 10.1111/j.1399-6576.2011.02646.x

[11] Lee A Ngan Kee WD Gin T A quantitative, systematic review of randomized controlled trials of ephedrine versus phenylephrine for the management of hypotension during spinal anesthesia for cesarean delivery. Anesth Analg 2002;94:920–6. 11916798 10.1097/00000539-200204000-00028

[12] Heesen M Kolhr S Rossaint R Prophylactic phenylephrine for caesarean section under spinal anaesthesia: systematic review and meta-analysis. Anaesthesia 2014;69:143–65. 24588024 10.1111/anae.12445

[13] Stewart A Fernando R McDonald S The dose-dependent effects of phenylephrine for elective cesarean delivery under spinal anesthesia. Anesth Analg 2010;111:1230–7. 20841418 10.1213/ANE.0b013e3181f2eae1

[14] Ngan Kee WD Lee SW Ng FF Randomized double-blinded comparison of norepinephrine and phenylephrine for maintenance of blood pressure during spinal anesthesia for cesarean delivery. Anesthesiology 2015;122:736–45. 25635593 10.1097/ALN.0000000000000601

[15] Shamseer L Moher D Clarke M Preferred reporting items for systematic review and meta-analysis protocols (PRISMA-P) 2015: elaboration and explanation. BMJ (Clinical research ed) 2015;350:g7647. 10.1136/bmj.g7647 25555855

[16] Higgins JPT, S G. Cochrane handbook for systematic reviews of interventions. Chichester, England: Wiley-Blackwell; 2008.

[17] Cornell JE The PRISMA extension for network meta-analysis: bringing clarity and guidance to the reporting of systematic reviews incorporating network meta-analyses. Ann Intern Med 2015;162:797–8. 26030637 10.7326/M15-0930

[18] Chaimani A Higgins JP Mavridis D Graphical tools for network meta-analysis in STATA. PloS One 2013;8:e76654. 24098547 10.1371/journal.pone.0076654 PMC3789683

[19] White IR Barrett JK Jackson D Consistency and inconsistency in network meta-analysis: model estimation using multivariate meta-regression. Res Synth Methods 2012;3:111–25. 26062085 10.1002/jrsm.1045 PMC4433771

[20] Salanti G Ades AE Ioannidis JP Graphical methods and numerical summaries for presenting results from multiple-treatment meta-analysis: an overview and tutorial. J Clin Epidemiol 2011;64:163–71. 20688472 10.1016/j.jclinepi.2010.03.016

[21] Riley RD Higgins JP Deeks JJ Interpretation of random effects meta-analyses. BMJ (Clinical research ed) 2011;342:d549. 10.1136/bmj.d549 21310794

