# Supplementary material for: Vasopressors for the management of maternal hypotension during cesarean section under spinal anesthesia: A Systematic review and network meta-analysis protocol
Source: Medicine (Baltimore). 2019 Jan 4;98(1):e13947. doi: 10.1097/MD.0000000000013947 (PMC6344174; doi:10.1097/MD.0000000000013947)
Supplement: Supplemental Digital Content [file medi-98-e13947-s001.docx]

**Appendix**

**Search terms for MEDLINE**

1. randomized controlled trial.pt
2. randomized controlled trial$.mp
3. controlled clinical trial.pt
4. controlled clinical trial$.mp
5. random allocation.mp
6. exp double-blind method/
7. double-blind.mp
8. exp single-blind method/
9. single-blind.mp
10. or/1-9
11. clinical trial.pt
12. clinical trial$.mp
13. exp clinical trial/
14. (clin$ adj25 trial$).mp
15. ((singl$ or doubl$ or tripl$ or trebl$) adj25 (blind$ or mask$)).mp
16. random$.mp
17. exp research design/
18. research design.mp
19. or/11-18
20. 10 or 19
21. Case report.tw
22. Letter.pt
23. Historical article.pt
24. Review.pt
25. or/21-24
26. 20 not 25
27. Exp Cesarean Section/
28. Caesarean section.mp
29. Cesarean delivery.mp
30. Cesarean deliver.mp.
31. C-section.mp
32. C section.mp
33. C-sections.mp
34. Abdominal delivery.mp
35. Abdominal deliveries.mp
36. Delivery, abdominal.mp
37. Or/27-36
38. Exp Anesthesia, spinal/
39. Spinal anesthesia.mp
40. Spinal anaesthesia.mp
41. Subarachnoid block.mp
42. Subarachnoid anesthesia.mp
43. Subarachinoid anaesthesia.mp
44. Axial anesthesia.mp
45. Axial anaestheia.mp
46. Exp Anesthesia, conduction/
47. Or/38-46
48. 37 and 47
49. 26 and 48
50. Exp vasoconstrictor agents/
51. Vasoconstrictor.mp
52. Vasopressor.mp
53. Vasoacitve.mp
54. Exp ephedrine/
55. Ephedrine.mp
56. Sal-phedrine/
57. Sal Phedrine/
58. Salphedrine
59. Ephedrine sulphate/
60. Ephedrine hydrochloride/
61. Ephedrine renaudin/
62. Exp metaraminol/
63. Meta-hydroxynorephedrine/
64. Metaradrin/
65. Isophenlyephrine/
66. Aramine/
67. Araminol/
68. Mephentermine/
69. Exp phenylephrine/
70. Phenylephrine.mp
71. Metasympatol/
72. Mezaton/
73. Metaoxedrine
74. Neo-Synephrine/
75. Neo Synephrine/
76. Neosynephrine/
77. Exp methoxamine/
78. Methoxamine.mp
79. Methoxamedrin/
80. Vasylox/
81. Vasoxine/
82. Vasoxin/
83. Vasoxyl/
84. Exp norepinephrine/
85. Norepinephrine/
86. Noradrenaline/
87. Levarterenol/
88. Levonorepinephrine/
89. Levonor/
90. Levophed/
91. Arterenol/
92. Exp epinephrine/
93. Epinephrine.mp
94. Adrenaline/
95. Epitrate/
96. Lyophrin/
97. Epifrin/
98. Or/50-97
99. 49 and 98

**Search terms for Embase**

1. randomi?ed controlled trial$.mp
2. 'controlled clinical trial (topic)'/exp
3. controlled AND clinical AND trials
4. controlled clinical trial$.mp
5. 'randomization'/exp
6. 'random allocation'/exp
7. random allocation.mp
8. double-blind.mp
9. single-blind.mp
10. #1 OR #2 OR #3 OR #4 OR #5 OR #6 OR #7 OR #8 OR #9
11. 'clinical trial (topic)'/exp
12. clinical AND trial$.mp
13. random$.mp
14. rct
15. #11 OR #12 OR #13 OR #14
16. #10 OR #15
17. 'case study'/exp
18. 'case report'/exp
19. 'abstract report'/exp
20. 'letter'/exp
21. #17 OR #18 OR #19 OR #20
22. #16 NOT #21
23. 'cesarean section'/exp
24. Caesarean section.mp
25. Cesarean delivery.mp
26. Cesaerean delivery.mp
27. C-section.mp
28. C section.mp
29. C-sections.mp
30. abdominal AND delivery.mp
31. #23 OR #24 OR #25 OR #26 OR #27 OR #28 OR #29 OR #30
32. 'spinal anesthesia'/exp
33. spinal AND anesthesia
34. spinal AND anaesthesia
35. subarachnoid AND block
36. Subarachnoid anesthesia
37. subarachinoid AND anaesthesia
38. axial AND anesthesia
39. axial AND anaestheia
40. #32 OR #33 OR #34 OR #35 OR #36 OR #37 OR #38 OR #39
41. #31 AND #40
42. #22 AND #41
43. 'hypertensive agent'/exp
44. Vasoconstrictor
45. Vasopressor
46. Vasoacitve
47. 'ephedrine'/exp
48. Ephedrine
49. Sal-phedrine
50. Sal Phedrine
51. Salphedrine
52. 'metaraminol'/exp
53. Meta-hydroxynorephedrine
54. Metaradrin
55. Isophenlyephrine
56. Aramine
57. Araminol
58. Mephentermine
59. 'phenylephrine'/exp
60. Phenylephrine
61. Metasympatol
62. Mezaton
63. Metaoxedrine
64. Neo-Synephrine
65. Neosynephrine
66. 'methoxamine'/exp
67. Methoxamine
68. Methoxamedrin
69. Vasylox
70. Vasoxine
71. Vasoxin
72. Vasoxyl
73. 'noradrenalin'/exp
74. Norepinephrine
75. Noradrenaline
76. Levarterenol
77. Levonorepinephrine
78. Levonor
79. Levophed
80. Arterenol
81. 'epinephrine'/exp
82. Epinephrine
83. Adrenaline
84. Epitrate
85. Lyophrin
86. Epifrin
87. #43 OR #44 OR #45 OR #46 OR #47 OR #48 OR #49 OR #50 OR #51 OR #52 OR #53 OR #54 OR #55 OR #56 OR #57 OR #58 OR #59 OR #60 OR #61 OR #62 OR #63 OR #64 OR #65 OR #66 OR #67 OR #68 OR #69 OR #70 OR #71 OR #72 OR #73 OR #74 OR #75 OR #76 OR #77 OR #78 OR #79 OR #80 OR #81 OR #82 OR #83 OR #84 OR #85 OR #86
88. #42 AND #87
